# Associations of *TFEB* Gene Polymorphisms With Cognitive Function in Rural Chinese Population

**DOI:** 10.3389/fnagi.2021.757992

**Published:** 2021-12-14

**Authors:** Yanfei Wei, Shuzhen Liu, Jiansheng Cai, Xu Tang, Junling Zhang, Min Xu, Qiumei Liu, Chunmei Wei, Xiaoting Mo, Shenxiang Huang, Yinxia Lin, Tingyu Mai, Dechan Tan, Tingyu Luo, Ruoyu Gou, Huaxiang Lu, Jian Qin, Zhiyong Zhang

**Affiliations:** ^1^Department of Occupational and Environmental Health, School of Public Health, Guangxi Medical University, Guangxi, China; ^2^Key Laboratory of Tumor Immunology and Microenvironmental Regulation, Guilin Medical University, Guilin, China; ^3^Key Laboratory of Longevity and Aging-Related Diseases of Chinese Ministry of Education, Nanning, China; ^4^Department of Environmental Health and Occupational Medicine, School of Public Health, Guilin Medical University, Guangxi, China

**Keywords:** cognitive function, *TFEB*, single nucleotide polymorphism, haplotype, autophagy

## Abstract

**Background:** The study aimed to investigate the relationship between transcription factor EB (*TFEB*) gene polymorphisms, including their haplotypes, and the cognitive functions of a selected population in Gongcheng County, Guangxi.

**Methods:** A case-control study approach was used. The case group comprised 339 individuals with cognitive impairment, as assessed by their Mini-Mental State Examination scores; the control population also comprised 339 individuals who were matched by sex and age (± 5 years) in a 1:1 ratio. *TFEB* gene polymorphisms were genotyped in 678 participants (190 men and 488 women, aged 30–91 years) by using the Sequenom MassARRAY platform.

**Results:** Multifactorial logistic regression analysis showed that in the dominant model, the risk of developing cognitive impairment was 1.547 times higher in cases with the *TFEB* rs14063A allele (AG + AA) than in those with the GG genotype (adjusted odds ratio [OR] = 1.547, Bonferroni correction confidence interval = 1.021–2.345). Meanwhile, the presence of the *TFEB* rs1062966T allele (CT + TT) was associated with a lower risk of cognitive impairment in comparison with the presence of the CC genotype (adjusted OR = 0.636, Bonferroni correction confidence interval = 0.405–0.998). In the co-dominant model, the risk of developing cognitive impairment was 1.553 times higher in carriers of the *TFEB* rs14063AG genotype than in carriers of the GG genotype (adjusted OR = 1.553, Bonferroni correction confidence interval = 1.007–2.397). After the Bonferroni correction and adjustment for confounding factors, the association of *TFEB* rs1062966 with cognitive function persisted in the analyses stratified by education level. Ethnically stratified analysis showed a significant association between *TFEB* rs1062966 and cognitive function in the Yao population. The multilocus linkage disequilibrium analysis indicated that the identified single nucleotide polymorphisms were not inherited independently. The haplotype analysis suggested that the rs14063A–rs1062966C–rs2278068C–rs1015149T haplotype of the *TFEB* gene increased the risk of cognitive impairment (*P* < 0.05) and that the rs14063G–rs1062966T–rs2278068C–rs1015149C haplotype was associated with a reduced risk of cognitive impairment (*P* < 0.05).

**Conclusion:** *TFEB* rs1062966 polymorphisms and their rs14063A–rs1062966C–rs2278068C–rs1015149T and rs14063G–rs1062966T–rs2278068C–rs1015149C haplotypes are genetic factors that may affect cognitive function among the rural Chinese population.

## Introduction

Cognitive impairment, which is also regarded as a neurocognitive disorder, typically affects learning ability, memory, perceptual–motor function, language, attention, and problem solving (Robbins et al., 2019). Cognitive impairment mainly includes mild cognitive impairment and dementia. Cognitive impairment is a growing public health problem; for example, about 38.77 million people in China have been recorded as having mild cognitive impairment (Jia et al., 2020), and every year, about 8%–15% of them progress to dementia (Petersen, 2016). Previous studies have shown that cognitive function is mainly influenced by lifestyle, some genetic polymorphisms, and diseases (Lv et al., 2017; Zaki et al., 2019; Dokkedal et al., 2020; Zhang et al., 2020). Therefore, the research on genetic susceptibility can help to screen vulnerable groups and improve preventive measures.

Neurodegenerative diseases, such as Alzheimer’s disease (AD), Parkinson’s disease (PD), and Huntington’s disease (HD) are associated with dysregulation of autophagy (Cortes and La Spada, 2019). Insoluble amyloid–β (Aβ) peptide deposition is one of the key hallmarks of AD pathology (Querfurth and LaFerla, 2010; Gouras et al., 2015). In recent years, numerous studies have shown that enhanced Aβ peptide clearance helps prevent the progression of AD (Selkoe and Hardy, 2016; Sevigny et al., 2016). The induction of the autophagy–lysosome pathway is an important therapeutic strategy for the clearance of Aβ peptides (Wani et al., 2019; Luo et al., 2020). Autophagy is a pathway of cellular self-digestion and is particularly important in protein metabolism in the central nervous system (CNS). It is also a key pathway for intracellular protein clearance, and the abnormal regulation of autophagy levels and alterations in autophagic pathway-related proteins play an important role in the pathogenesis of various diseases related to neurological cognitive dysfunction (Xiao et al., 2015; Bao et al., 2016; Torra et al., 2018). Transcription factor EB (*TFEB*) is a master regulator for the transcription of genes involved in autophagy and lysosomal biogenesis (Settembre et al., 2011; Martini-Stoica et al., 2016) as it promotes the expression of genes required for autophagosome formation, lysosome biogenesis, and lysosome function. It is highly expressed in CNS. *TFEB* overexpression has been proved to ameliorate the progression of neurodegenerative diseases, including PD, HD, and AD, as well as other tauopathy diseases. A growing body of evidence suggests that protein aggregation and autophagy and/or lysosomal dysfunction are the main pathogenic mechanisms in such diseases (Menzies et al., 2015). In addition, *TFEB* induces the intracellular clearance of pathogenic factors in a variety of murine models of diseases, such as PD and AD (Napolitano and Ballabio, 2016). Studies have revealed that *TFEB* is implicated in the pathogenesis of many neurodegenerative diseases. However, the effects of *TFEB* gene polymorphisms on cognitive function in populations at home and abroad have not been reported.

Guangxi Gongcheng Yao Autonomous County is located in northeastern Guangxi and is characterized by a spatially aggregated population distribution, with minimal population movement and consistent lifestyle and environment. Hence, it is conducive to the study of the effects of genetic and environmental factors on cognitive function. In the present study, the cognitive function of the population in Guangxi Gongcheng County was assessed on the basis of the Mini-Mental State Examination (MMSE). In addition, the polymorphisms and haplotypes of the *TFEB* gene were analyzed by determining the *TFEB* gene polymorphisms, and the relationship between cognitive function and the *TFEB* gene among the Guangxi Gongcheng County population was preliminarily explored. The results of the study are expected to provide new ideas and strategies for the tertiary prevention of cognitive dysfunction in the population on the basis of the mastery of the association between cognitive function and the *TFEB* gene.

## Materials and Methods

### Study Populations

In this case–control study, the study population was drawn from the residents of Gongcheng County, Guangxi Zhuang Autonomous Region, who participated in a health survey in December 2018–December 2019. The following criteria were observed: (a) residents of the study area; (b) individuals who did not suffer from psychosis or other neurological disorders that may cause cognitive impairment, such as epilepsy, schizophrenia, alcoholism, and nervous system tumor; (c) individuals who did not suffer from severe visual or auditory impairment; (d) individuals who had not taken medication that could severely affect neurological function within the last 2 weeks. Then, 339 individuals with cognitive impairment according to the MMSE results were selected as the case group. The control population was matched by sex and age (± 5 years) at a 1:1 ratio. A total of 678 individuals aged 30–91 years were included. The research protocol was approved by the medical ethical committee of Guilin Medical University. All participants provided written informed consent.

### Epidemiological Survey and Biochemical Measurements

Under the principle of informed consent, a standardized and uniform questionnaire was used. A face–to–face conversational questionnaire was administered to the study subjects by investigators who had received uniform and rigorous training. The main content of the questionnaire included information on demographics, past medical history, socioeconomic status, lifestyle, and cognitive functional status. Height (measured with a rangefinder with accuracy of up to 0.1 cm), weight (the participants were weighed with minimal clothing and with shoes off; accuracy was up to 0.1 kg), and waist circumference were measured as part of the physical examination. Body mass index (BMI) was calculated from the weight and height data, i.e., weight (kg)/height^2^ (m^2^). Fasting venous blood was taken on the day of the physical examination and transported to the Laboratory Department of Gongcheng Yao Autonomous County People’s Hospital through.

cold chain transportation method in the morning of the same day. Fasting blood glucose (FPG) was tested by a blood routine analyzer (Sysmex CS–1600, Shanghai, China). The concentrations of glycosylated hemoglobin, uric acid (UA), serum total cholesterol (TC), high–density lipoprotein cholesterol (HDL–C), low-density lipoprotein cholesterol (LDL–C), and serum triglycerides (TG) were determined using a blood biochemical detector (Hitachi 7600–020, Kyoto, Japan).

### Cognitive Function Assessment

Cognitive function assessment was assessed by face-to-face interviews using the Chinese version of the MMSE. The MMSE scale is the most commonly used tool to screen for cognitive impairment in clinical, research, and community settings (Folstein et al., 1975; Ismail et al., 2010; Arevalo-Rodriguez et al., 2015) and is widely used in large–scale epidemiological studies of dementia with high validity and reliability (Cao et al., 2012; Bickel et al., 2018). MMSE assesses five dimensions, namely, orientation (10 points), attention and calculation (5 points), memory (6 points), language skills (8 points), and visual–spatial (1 point) for a total score of 30 points. High MMSE scores indicate good cognitive function. Several studies have proved that education exerts an impact on cognitive function (Crum et al., 1993; Li H. et al., 2016; Lovden et al., 2020). Given the strong influence of education on cognitive function, we used the MMSE score cut–off points for different literacy levels to determine whether the study participants suffered from cognitive decline. The cognitive impairment group was divided on the basis of a cut-off point of 16/17 (MMSE score < 17) for the participants with no formal education, 19/20 (MMSE score < 20) for those with 1–6 years of education, and 23/24 (MMSE score < 24) for those with > 6 years of education (Li H. et al., 2016); the rest belonged to the cognitively normal group.

### Single Nucleotide Polymorphism Selection and Genotyping

A two--part locus screening strategy involving functional single nucleotide polymorphisms (SNPs) and validated SNPs, was used to screen for the important candidate gene *TFEB*. The strategy was as follows: (1) Functional regional SNP screening: The *TFEB* gene was searched in the NCBI-SNP^1^ website, and the *TFEB* gene promoter (upstream variant 2KB), 5′UTR, Exon (missense, synonymous), and functional SNP loci in the 3′UTR region (the relevant optimization parameter was minor allele frequency (MAF) in CHB > 0.05 according to HapMap or 1000Genomes database). The relevant literature was checked, and the results with disease susceptibility were annotated. The screened SNP loci were used for functional prediction using the NIH website^2^. The SNP loci screened by^3^ were used to perform linkage disequilibrium (LD) analysis on the SNP loci screened in the first step and annotate the fully linked loci with R (Jia et al., 2020) = 1. (2) Validated and hot SNP screening: SNP loci with susceptibility were screened via a Google Scholar search, and then the screened SNP loci were verified before functional prediction at *http://snpinfo.niehs.nih.gov/.* Finally, the SNP loci of the appealing candidate genes was optimized by discarding the loci with R (Jia et al., 2020) = 1 and keeping the loci with R (Jia et al., 2020) > 0.8 in the promoter region. After the appeal screening, rs1015149, rs1062966, rs14063, rs2273068, rs11754668, and rs73733015 loci of the *TFEB* gene for typing were screened.

Blood samples were drawn from the participants by a professional nurse. DNA was extracted from 1 mL of blood samples by using the Blood DNA Kit (Tiangen, Beijing, China). Genotyping of SNPs was conducted, and primer design and synthesis were performed by Bio Miao Biological Technology Co., Ltd. (Beijing, China) by using the Sequenom MassARRAY matrix–assisted laser desorption ionization time–of–flight mass spectrometry platform (Sequenom Inc., San Diego, CA, United States). The primer sequence information is shown in Supplementary Table 1. PCR (ABI) was used to amplify the DNA. A 384–well SpectroCHIP bioarray was used as the microarray.

### Statistical Analysis

The data were statistically analyzed using SPSS22.0 statistical software. Normally distributed quantitative data were expressed in terms of mean ± SD, and the t-test of independence was used for comparison between groups. MMSE scores were not normally distributed and expressed as median and interquartile range, and comparison between groups was performed via the Wilcoxon or Kruskal–Wallis rank sum test. The qualitative data were presented in terms of percentage and were analyzed for the control and cognitive impairment groups by using a chi-square test or Fisher’s exact probability method. After adjusting for gender, age, education, and ethnicity as covariates, a multifactorial logistic regression model was used to calculate the odds ratio (OR) and 1–α confidence interval (CI) for assessing the association between the SNPs and cognitive function. A Bonferroni corrected p-value was applied to the multifactorial logistic regression *p*–values to account for the multiple testing of six different SNPs in the same samples (corrected α = 0.05/6 = 0.00833). LINK 1.90 software was used for the analysis of the Hardy--Weinberg equilibrium (HWE) and MAF statistics for the study population. Pair--wise LD and haplotype frequencies among the SNPs were analyzed using Haploview (Broad Institute of MIT and Harvard, United States, version 4.2). The analysis of the haplotypes comprising strongly linked SNP loci and risk of cognitive impairment was performed using SHEsis Main online software^4^. The criterion for significance was set at *P* < 0.05 for all tests.

## Results

### Characteristics of Studied Population

The demographic parameters of the 678 study subjects are summarized in Table 1, where the median MMSE score was 21 and the interquartile range was 17–25. No statistically significant differences in mean age, sex ratio, BMI, ethnicity, smoking status, and alcohol consumption between the control and cognitively impaired groups (*P* > 0.05) were observed. In addition, a significant difference in the distribution of different education levels between the two groups (*P* < 0.05) was noted.

**TABLE 1:** Comparison of general characteristics of control and cognitive impairment groups.

Parameter	Total	Control	Cognitive impairment	t/χ^2^	P
Number	678	339	339		
MMSE scores	21.00 (17.00–25.00)	25.00 (23.00–28.00)	17.00 (14.00–19.00)	−20.653	<0.001
Age (years) ^a^	64.18 ± 10.78	63.72 ± 10.75	64.63 ± 10.80	−1.105	0.270
Body mass index (kgm^2^)	22.15 ± 3.41	22.20 ± 3.26	22.09 ± 3.57	0.382	0.703
Male/female ^b^	190/488	95/244	95/244	0.000	1.000
Ethnicity, *n* (%) Yao minority Others	470 (69.32) 208 (30.68)	237 (69.91) 102 (30.09)	233 (68.73) 106 (31.27)	0.111	0.739
Current smoker, *n* (%) Yes No	95 (14.01) 583 (85.99)	45 (13.27) 294 (86.73)	50 (14.75) 289 (85.25)	0.306	0.580
Current drinker, *n* (%) Yes No	192 (28.32) 486 (71.68)	86 (25.37) 253 (74.63)	106 (31.27) 233 (68.73)	2.906	0.088
Education level, *n* (%) No formal educated 1–6 years of education 7 or more years of education	382 (56.34) 132 (19.47) 164 (24.19)	166 (48.97) 86 (25.37) 87 (25.66)	216 (63.72) 46 (13.57) 77 (22.71)	19.275	<0.001

*^a^Mean ± SD was detected by t-test; ^b^Differences in the classified data were determined in a chi-square test.*

### Genotype and Allele Frequencies

The SNP loci rs1015149, rs1062966, rs11754668, rs14063, rs2273068, and rs73733015 of the *TFEB* gene were successfully typed. Hence, all the above loci were located on chromosome 6. The MAFs of each locus were all greater than 0.05, which indicated non-low frequency variants. The genotype distribution of six SNPs in the cognitive impairment and control groups met the HWE (*P*_HWE_ > 0.05 for all). Table 2 shows that the allele and genotype frequencies of the *TFEB* rs1062966 and rs14063 SNPs in this study differed between the control and cognitive impairment groups (*P* < 0.05). The frequency of *TFEB* rs73733015C allele was higher in the cognitive impairment group than in the control group, and the difference was statistically significant between the two groups (*P* < 0.05).

**TABLE 2:** Genotypic and allelic frequencies of *TFEB* SNPs in the control and cognitive impairment groups [*n* (%)].

SNP	Genotype (allele)	Total (*N* = 678)	Control (*N* = 339)	Cognitive impairment (*N* = 339)	χ^2^	P
rs1015149 C > T	CC CT TT C T MAF P_HWE_	231 (34.07) 324 (47.79) 123 (18.14) 786 (57.96) 570 (42.04) 0.6363	123 (36.28) 164 (48.38) 52 (15.34) 410 (60.47) 268 (39.53) 0.9096	108 (31.86) 160 (47.20) 71 (20.94) 376 (55.46) 302 (44.54) 0.4415	3.958 3.499	0.138 0.061 0.4204
rs1062966 C > T	CC CT TT C T MAF P_HWE_	470 (69.32) 191 (28.17) 17 (2.51) 1,131 (83.41) 225 (16.59) 0.7811	220 (64.90) 108 (31.86) 11 (3.24) 548 (80.83) 130 (19.17) 0.7267	250 (73.75) 83 (24.48) 6 (1.77) 583 (85.99) 95 (14.01) 1	6.658 6.528	0.036 0.011 0.1659
rs11754668 C > G	CC GC GG C G MAF P_HWE_	571 (84.22) 103 (15.19) 4 (0.59) 1,245 (91.81) 111 (8.19) 1	290 (85.55) 47 (13.86) 2 (0.59) 627 (92.48) 51 (7.52) 1	281 (82.89) 56 (16.52) 2 (0.59) 618 (91.15) 60 (8.85) 1	0.928 0.795	0.629 0.375 0.08186
rs14063 G > A	GG AG AA G A MAF P_HWE_	330 (48.67) 294 (43.36) 54 (7.96) 954 (70.35) 402 (29.65) 0.3569	181 (53.39) 133 (39.23) 25 (7.38) 495 (73.01) 183 (26.99) 0.8913	149 (43.95) 161 (47.50) 29 (8.55) 459 (67.70) 219 (32.30) 0.1362	6.066 4.582	0.048 0.032 0.2965 0.061
rs2273068 C > T	CC CT TT C T MAF P_HWE_	543 (80.09) 130 (19.17) 5 (0.74) 1,216 (89.68) 140 (10.32) 0.531	273 (80.53) 63 (18.58) 3 (0.89) 609 (89.82) 69 (10.18) 1	270 (79.65) 67 (19.76) 2 (0.59) 607 (89.53) 71 (10.47) 0.5577	0.340 0.032	0.844 0.858 0.1032
rs73733015 C > G	CC CG GG C G MAF P_HWE_	433 (63.86) 219 (32.30) 26 (3.83) 1,085 (80.01) 271 (19.99) 0.9044	204 (60.18) 119 (35.10) 16 (4.72) 527 (77.73) 151 (22.27) 0.8762	229 (67.55) 100 (29.50) 10 (2.95) 558 (82.30) 120 (17.70) 1	4.476 4.432	0.107 0.035 0.1999

*Qualitative data were assessed using the chi-square test; P < 0.05 indicated a statistically significant difference; P_HWE_ > 0.05 indicated a statistically significant difference.*

### Genotype and Allele Frequencies and Their Respective Associations With Cognitive Impairment

The associations of the six SNPs with cognitive impairment are shown in Table 3. Using multifactorial logistic regression, the associations of SNP locus genotypes and four SNP genetic models with cognitive impairment were analyzed. After Bonferroni correction and adjustment for age, gender, ethnicity, and education level as covariates, the association of *TFEB* rs1062966 and rs14063 SNPs with cognitive impairment was significant in the dominant model in the total population (*P* < 0.00833). Subjects carrying the *TFEB* rs1062966T allele (CT + TT) showed a lower risk of cognitive impairment than the subjects with the CC genotype (dominant model: adjusted OR = 0.636, Bonferroni correction confidence interval = 0.405–0.998, *P* = 0.008). The risk of developing cognitive impairment was 1.547 times higher in those carrying the *TFEB* rs14063A allele (AG + AA) than in those with the GG genotype (dominant model: adjusted OR = 1.547, Bonferroni correction confidence interval = 1.021–2.345, *P* = 0.006). In the co-dominant model, subjects carrying the *TFEB* rs14063AG genotype had an increased risk of cognitive impairment relative to those with the GG genotype, and the risk of cognitive impairment was 1.553 times higher in the AG carriers than in the GG carriers (co-dominant model: adjusted OR = 1.553, Bonferroni correction confidence interval = 1.007–2.397, *P* = 0.007). The association between genotype and cognitive functional status for the remaining SNP loci was not statistically significant.

**TABLE 3:** Associations between genetic models of four SNPs and cognitive impairment.

SNP	Model	Genotype		Adjusted OR (confidence interval)	* *P*
		Reference	Alternate		
rs1015149 C > T	Co-dominant Dominant Recessive Overdominant	CC CC CC + CT CC + TT	CT TT CT + TT TT CT	1.156 (0.727-1.838) 1.653 (0.903-3.024) 1.276 (0.825-1.972) 1.519 (0.885-2.608) 0.971 (0.642-1.469)	0.409 0.028 0.141 0.041 0.852
rs1062966 C > T	Co-dominant Dominant Recessive Overdominant	CC CC CC + CT CC + TT	CT TT CT + TT TT CT	0.657 (0.413-1.045) 0.436 (0.110-1.723) 0.636 (0.405-0.998) 0.493 (0.125-1.936) 0.677 (0.427-1.073)	0.017 0.111 **0.008** 0.172 0.025
rs11754668 C > G	Co-dominant Dominant Recessive Overdominant	CC CC CC + GC CC + GG	GC GG GC + GG GG GC	1.249 (0.701-2.227) 0.845 (0.059-12.082) 1.232 (0.698-2.174) 0.817 (0.057^–^11.671) 1.251 (0.702-2.228)	0.310 0.868 0.332 0.841 0.307
rs14063 G > A	Co-dominant Dominant Recessive Overdominant	GG GG GG + AG GG + AA	AG AA AG + AA AA AG	1.553 (1.007-2.397) 1.514 (0.686-3.340) 1.547 (1.021-2.345) 1.238 (0.577-2.656) 1.464 (0.964-2.225)	**0.007** 0.167 **0.006** 0.460 0.016
rs2273068 C > T	Co-dominant Dominant Recessive Overdominant	CC CC CC + CT CC + TT	CT TT CT + TT TT CT	1.065 (0.631-1.796) 0.705 (0.061-8.170) 1.049 (0.627-1.756) 0.697 (0.060-8.052) 1.068 (0.633-1.801)	0.752 0.707 0.807 0.697 0.739
rs73733015 C > G	Co-dominant Dominant Recessive Overdominant	CC CC CC + CG CC + GG	CG GG CG + GG GG CG	0.757 (0.485-1.182) 0.556 (0.184-1.684) 0.733 (0.477-1.126) 0.611 (0.204-1.830) 0.782 (0.503-1.216)	0.099 0.162 0.056 0.236 0.142

Analysis was conducted after adjustment for covariates, including age, gender, education level, and ethnicity. OR, odds ratio; confidence interval, Bonferroni correction confidence interval; *P-value < 0.00833 indicated a statistically significant difference after Bonferroni correction (shown in bold; 6 SNPs = 6 tests).

Given the effect of education level on cognitive function, the study population was further stratified into three groups for analysis in accordance with the number of years of formal education received. As shown in Supplementary Table 2, after Bonferroni correction and adjustment for gender, age and ethnicity, results showed a significant association between *TFEB* rs1062966 and cognitive function in the group with no formal education. In the co-dominant model, the presence of the *TFEB* rs1062966 CT genotype was more strongly associated with a reduced risk of cognitive impairment than the presence of the CC genotype (co-dominant model: adjusted OR = 0.510, Bonferroni correction confidence interval = 0.275–0.948, *P* = 0.004). In the dominant model, subjects carrying the *TFEB* rs1062966T allele (CT + TT) had a lower risk of cognitive impairment than those carrying the CC genotype (dominant model: adjusted OR = 0.510, Bonferroni correction confidence interval = 0.281–0.928, P = 0.003). In the overdominant model, the presence of the *TFEB* rs1062966CT genotype was more closely associated with a reduced risk of cognitive dysfunction than the presence of the CC + TT genotype (overdominant model: adjusted OR = 0.527, Bonferroni correction confidence interval = 0.285–0.974, *P* = 0.006). The remaining SNP loci were not statistically associated with cognitive function in any of the four genetic models (*P* > 0.0833).

Ethnic differences in genetic background might exist in different racial/ethnic groups. Therefore, the study population was further stratified by ethnicity for analysis. As shown in Supplementary Table 3, after Bonferroni correction and adjustment for sex, age, and education level, results showed a significant association between *TFEB* rs1062966 and cognitive function in the Yao minority group. In the co-dominant model, subjects carrying the CT genotype had a lower risk of cognitive impairment than those carrying the CC genotype (co-dominant model: adjusted OR = 0.563, Bonferroni correction confidence interval = 0.317–0.999, P = 0.008). In the dominant model, subjects carrying the *TFEB* rs1062966T allele (CT + TT) showed a lower risk of cognitive impairment than those carrying the CC genotype (dominant model: adjusted OR = 0.543, Bonferroni correction confidence interval = 0.311–0.946, *P* = 0.004). In other ethnic groups, SNP loci were not statistically different from cognitive function in any of the four genetic models (*P* > 0.0833).

### Haplotypes and the Risk of Cognitive Impairment

**FIGURE 1:**
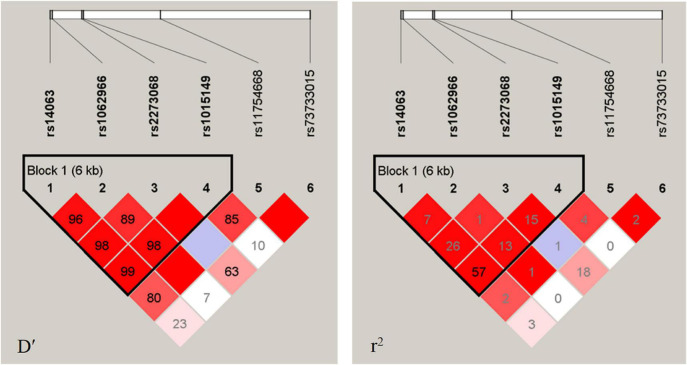
LD analysis of the *TFEB* SNPS in both populations. The LD degree was represented by pair-wise D′ and r^2^. The strength of LD is reflected by the color, and the correlation of LD increases as the color increases, with white being the weakest and crimson being the strongest.

Figure 1 shows the LD analysis of the SNPs of *TFEB*. Multilocus LD analyses indicated that the tested sites in the study population were not statistically independent and that the control and cognitive impairment groups showed strong LD (D’ = 0.89–0.99). As shown in Table 4, the dominant haplotypes were rs14063G–rs1062966C–rs2278068C–rs1015149C (>40% of the samples) and rs14063A–rs1062966C–rs2278068C–rs1015149T (>20% of the samples). The rs14063A–rs1062966C–rs2278068C–rs1015149T and rs14063G–rs1062966T–rs2278068C–rs1015149C haplotype frequencies were statistically different between the two groups (*P* < 0.05 for all). The rs14063A–rs1062966C–rs2278068C–rs1015149T haplotype was associated with an increased risk of cognitive impairment (OR = 1.370, 95% CI = 1.044–1.797, *P* = 0.022797), whereas the rs14063G–rs1062966T–rs2278068C–rs1015149C haplotype.

**TABLE 4:** Prevalence of haplotype frequencies in the cognitive impairment and control groups [*n* (frequency)].

Haplotype	Control (*N* = 678)	Cognitive impairment (*N* = 678)	χ^2^	*P*	OR (95% CI)
G–C–C–C	280.13 (0.413)	281.03 (0.414)	0.001	0.972127	0.996 (0.802–1.237)
A–C–C–T	114.18 (0.168)	148.00 (0.218)	5.186	0.022797	1.370 (1.044–1.797)
G–T–C–C	128.73 (0.190)	94.97 (0.140)	6.327	0.011908	0.690 (0.517–0.922)
G–C–C–T	84.79 (0.125)	82.99 (0.122)	0.035	0.852634	0.970 (0.702–1.340)
A–C–T–T	66.45 (0.098)	70.98 (0.105)	0.139	0.708808	1.070 (0.751–1522)

*The haplotype was combined with TFEB rs14063–rs1062966–rs2278068–rs1015149. Rare Hap (frequency < 3%) in both groups was ignored in the analysis; OR, odds ratio; CI, confidence interval; P < 0.05 indicated a statistically significant difference; n = total number with that haplotype.*

Was protective against cognitive function (OR = 0.690, 95% CI = 0.517–0.922, *P* = 0.011908).

## Discussion

To our knowledge, our findings suggest for the first time that *TFEB* gene polymorphisms affect cognitive function. The main findings of the current research included the following aspects (1) *TFEB* rs1062966 SNP and rs14063G–rs1062966T–rs2278068C–rs1015149C haplotypes in the total population’s *TFEB* gene are associated with a reduced risk of cognitive impairment, whereas rs14063 SNP and rs14063A–rs1062966C–rs2278068C–rs1015149T haplotypes increase the risk of cognitive impairment. (2) The stratified analysis of education level shows that in the population with no formal education, the *TFEB* rs1062966 SNP is significantly associated with cognitive function. (3) Ethnically stratified analysis shows a significant association between *TFEB* rs1062966 SNP and cognitive function was observed in the Yao population.

*TFEB* is a basic helix–loop–helix leucine zipper transcription factor. To our knowledge, cognitive decline may be associated with an imbalance in the production and clearance of β-amyloid. *TFEB* is a major regulator of the lysosomal pathway, and lysosomal dysfunction is speculated to have a central role in enhanced Aβ generation (Lee et al., 2010; Neely et al., 2011; Coen et al., 2012; McBrayer and Nixon, 2013) or its impaired clearance (Majumdar et al., 2011; Lee et al., 2012). Therefore, one of the possible explanations for the effect of *TFEB* on cognitive function in the present study is that *TFEB* is a master inducer of lysosomal biogenesis (Settembre et al., 2011), especially in astrocytes. It stimulates lysosomal biogenesis and function, accelerates Aβ uptake and degradation, and significantly reduces Aβ levels (Xiao et al., 2014). *TFEB* promotes autophagosome–lysosomal fusion (Palmieri et al., 2011) to attenuate the effective trafficking of Aβ production. In addition, *TFEB* can dynamically regulate ALP by coordinating autophagy induction with enhanced lysosomal clearance (Polito et al., 2014). In response to AD, ALP has been shown to regulate amyloid precursor protein (APP) turnover and Aβ metabolism (Nixon and Yang, 2011; Harris and Rubinsztein, 2012), and enhanced *TFEB* function can stimulate ALP function and promote protein clearance and neuroprotection (Sardiello et al., 2009; Tsunemi et al., 2012).

Other studies found that exogenous *TFEB* expression stimulates lysosomal biogenesis *in vitro*, reduces APP full length and its shear fragments, attenuates Aβ production and release, and significantly shortens the half-life of APP in a lysosome-dependent manner (Xiao et al., 2015). The aberrant expression or degradation APP is associated with AD. Therefore, as a strategy to reduce Aβ production, accelerating the degradation of the whole APP in lysosomes is effective. The levels of the APP degradation product sAPPα are correlated with MMSE scores (Rosen et al., 2014; Yun et al., 2020) and sAPPα functions to protect synaptic plasticity and learning memor*^y^* (Taylor et al., 2008). This finding is consistent with our finding that *TFEB* rs1062966SNP plays a protective role in cognitive function.

Furthermore, evidence suggests that the Tau protein hyperphosphorylation results in the formation of neurofibrillary tangles, which affect cognitive function (Mun et al., 2016), and *TFEB* can prevent cognitive impairment by selectively targeting the pathological tau species for clearance through the potent activation of multiple cellular degradative pathways (Polito et al., 2014; Wang et al., 2016; Chandra et al., 2018; Song et al., 2020; Xu et al., 2020). Moreover, a negative correlation has been observed between tau protein concentration and MMSE scores (Chen et al., 2019; Obrocki et al., 2020). Another study found that neuronal *TFEB* expression also reduces the half-life of Aβ (Xiao et al., 2015). *TFEB* transduction in neurons may stimulate Aβ uptake through macropinocytosis (Holmes et al., 2013). The findings of these studies suggested a beneficial role for *TFEB* in cognitive function. However, increased APP endocytosis by *TFEB* activation reportedly leads to enhanced β- and γ-cleavage, thereby accelerating Aβ production (Schneider et al., 2008; Xiao et al., 2012). This finding may explain why some individuals are at lower risk of cognitive impairment than others, whereas the predisposition of some persons to suffer cognitive deficits is due to autophagy impairment. In addition, the *TFEB* rs1062966 SNP is found to be associated with cognitive function in the Yao population in present study. Our results support previous research on racial/ethnic differences in cognition (Li M. et al., 2016; Tsai, 2018). This finding may be because different ethnic groups have different genetic backgrounds, or may be because the *TFEB* gene polymorphism is only part of a polygenic pattern that, together with environmental factors, acts in combination on cognitive function. Further investigation needs to be done in a large sample size. The association between *TFEB* gene polymorphisms and cognitive impairment observed in the present study indicates that polymorphisms at some SNP loci of the *TFEB* gene may affect *TFEB* gene expression and cognitive function. However, the exact mechanism of the association between *TFEB* gene polymorphisms and cognitive dysfunction is not clear. This issue may be explained at the molecular level by studying *TFEB* polymorphisms.

The following are the advantages of this study: (1) The study subjects are all from the same region with minimal differences in living environment and habits; these characteristics helped control the confounding factors. (2) On the basis of mastering the association between cognitive function and *TFEB* genes, new therapeutic targets for cognitive disorders may be provided. (3) Identifying the differences in *TFEB* mutation frequencies in the populations of the rural areas of Guangxi may provide new data for human genome research. The following are the potential limitations of this study: (1) One of the limitations of present study is its small sample size. Nevertheless, this study, being the first on *TFEB* gene polymorphism and cognitive function, may lay the foundation for future studies involving large samples. (2) Although the effect of the six SNPs of *TFEB* on cognitive function were detected, many potentially cognitive function-related SNPs were overlooked in the current study. (3) Although the association of *TFEB* SNPs with cognitive function in the present study was detected, many unmeasured environmental and genetic factors still need to be considered.

## Conclusion

In conclusion, *TFEB* rs1062966 polymorphisms and their rs14063A–rs1062966C–rs2278068C–rs1015149T and rs14063G–rs1062966T–rs2278068C–rs1015149C haplotypes are genetic factors that may affect cognitive function among the rural Chinese population. The findings must be further investigated by prospective studies with large samples.

## Data Availability Statement

The original contributions presented in the study are included in the article/Supplementary Material, further inquiries can be directed to the corresponding author/s.

## Ethics Statement

The studies involving human participants were reviewed and approved by the Medical Ethical Committee of Guilin Medical University. The patients/participants provided their written informed consent to participate in this study.

## Author Contributions

YW and SL analyzed the data and wrote the manuscript. JC analyzed the data and revised the manuscript. XT designed the study and reviewed the manuscript. JZ, MX, QL, CW, XM, SH, YL, TM, DT, TL, RG, and HL collected the relevant data. ZZ and JQ put forward the study topic and provided advice on the writing of the manuscript. All authors read and approved the final manuscript.

## Conflict of Interest

The authors declare that the research was conducted in the absence of any commercial or financial relationships that could be construed as a potential conflict of interest.

## Publisher’s Note

All claims expressed in this article are solely those of the authors and do not necessarily represent those of their affiliated organizations, or those of the publisher, the editors and the reviewers. Any product that may be evaluated in this article, or claim that may be made by its manufacturer, is not guaranteed or endorsed by the publisher.

## Footnotes

1
http://www.ncbi.nlm.nih.gov/snp

2
http://snpinfo.niehs.nih.gov/

3
http://asia.ensembl.org/Homo_sapiens/Tools/LD?db=cor

4
http://analysis.bio-x.cn/myAnalysis.php
