# Supplementary material for: Associations of TFEB Gene Polymorphisms With Cognitive Function in Rural Chinese Population
Source: Front Aging Neurosci. 2021 Dec 14;13:757992. doi: 10.3389/fnagi.2021.757992 (PMC8713571; doi:10.3389/fnagi.2021.757992)
Supplement: Supplementary file 1 [file Table_1.docx]

TABLE S1 | Primer sequence information of SNPs of *TFEB* gene.

| SNPs | Primer Sequence |
| --- | --- |
| rs1015149 | Forward: ACGTTGGATGATGGCGTCACGCATAGGGTT |
|  | Reverse: ACGTTGGATGTAATGCATGACAGCCTGTTG |
|  | UEP-SEQ: CTGGTGCTGCATGCGCTCCCG |
| rs1062966 | Forward: ACGTTGGATGTGAGGTCCCTGACCCTGAG |
|  | Reverse: ACGTTGGATGCTGAAGTCCAGGTGATGGAA |
|  | UEP-SEQ: CCATTCCAGGTGATGGAATGGGGA |
| rs11754668 | Forward: ACGTTGGATGTTCCAACAGCCCACCAAGTC |
|  | Reverse: ACGTTGGATGTTTCATACCTGGGTGTGGGC |
|  | UEP-SEQ: CACTCACTCCATCCG |
| rs14063 | Forward: ACGTTGGATGTTGGACTTAGTGCCTGTCTG |
|  | Reverse: ACGTTGGATGTGAAGGCCTCTTCCCACTG |
|  | UEP-SEQ: GGGACACTGCGCCAGTCAA |
| rs2273068 | Forward: ACGTTGGATGATCCCACATCCTACCATCTG |
|  | Reverse: ACGTTGGATGGGCTGATGTGGGCAGCAAA |
|  | UEP-SEQ: GTATTTGGGCAGCAAACTTGTT |
| rs73733015 | Forward: ACGTTGGATGTCAGCTCTGCACTTGTGCTC |
|  | Reverse: ACGTTGGATGTGCTCAACAAAGAAAGGCAG |
|  | UEP-SEQ: GGGGACAAAGAAAGGCAGCACTCATT |

Forward: upstream primer, Reverse: downstream primer, UEP-SEQ: single base extension primer.
