# Supplementary material for: Associations of TFEB Gene Polymorphisms With Cognitive Function in Rural Chinese Population
Source: Front Aging Neurosci. 2021 Dec 14;13:757992. doi: 10.3389/fnagi.2021.757992 (PMC8713571; doi:10.3389/fnagi.2021.757992)
Supplement: Supplementary file 2 [file Table_2.docx]

TABLE S2 | Associations between genetic models of four SNPs and cognitive impairment, stratified by different educational levels.

| SNP | Model | Genotype | | No formal educated | | 1–6 years of education | | 7 or more years of education | |
| --- | --- | --- | --- | --- | --- | --- | --- | --- | --- |
|  |  | Reference | Alternate | Adjusted OR  (confidence interval) | ^*^P | Adjusted OR  (confidence interval) | ^*^P | Adjusted OR  (confidence interval) | ^*^P |
| rs1015149 C > T | Co-dominant | CC | CT | 1.266 (0.693–2.313) | 0.302 | 0.697 (0.217–2.235) | 0.413 | 1.261 (0.473–3.364) | 0.533 |
|  |  |  | TT | 1.756 (0.762–4.047) | 0.075 | 0.861 (0.194–3.817) | 0.791 | 2.186 (0.678–7.046) | 0.078 |
|  | Dominant | CC | CT + TT | 1.373 (0.776–2.429) | 0.142 | 0.736 (0.243–2.230) | 0.466 | 1.527 (0.623–3.740) | 0.213 |
|  | Recessive | CC + CT | TT | 1.534 (0.719–3.272) | 0.137 | 1.096 (0.307–3.914) | 0.850 | 1.952 (0.682–5.590) | 0.093 |
|  | Overdominant | CC + TT | CT | 1.065 (0.615–1.845) | 0.761 | 0.736 (0.271–1.997) | 0.418 | 0.935 (0.388–2.251) | 0.840 |
| rs1062966 C > T | Co-dominant | CC | CT | 0.510 (0.275–0.948) | 0.004 | 1.287 (0.386–4.296) | 0.580 | 0.749 (0.298–1.887) | 0.410 |
|  |  |  | TT | 0.504 (0.097–2.607) | 0.272 | – | – | 0.219 (0.008–5.747) | 0.220 |
|  | Dominant | CC | CT + TT | 0.510 (0.281–0.928) | 0.003 | 1.150 (0.348–3.801) | 0.758 | 0.699 (0.282–1.731) | 0.297 |
|  | Recessive | CC + CT | TT | 0.607 (0.119–3.085) | 0.418 | – | – | 0.270 (0.011–6.557) | 0.279 |
|  | Overdominant | CC + TT | CT | 0.527 (0.285–0.974) | 0.006 | 1.290 (0.384–4.327) | 0.579 | 0.819 (0.325–2.062) | 0.568 |
| rs11754668 C > G | Co-dominant | CC | GC | 1.421 (0.661–3.054) | 0.225 | 1.340 (0.381–4.716) | 0.539 | 1.048 (0.256–4.289) | 0.929 |
|  |  |  | GG | 0.402 (0.016–10.345) | 0.459 | – | – | – | – |
|  | Dominant | CC | GC + GG | 1.336 (0.635–2.814) | 0.304 | 1.341 (0.381–4.723) | 0.538 | 1.198 (0.307–4.677) | 0.726 |
|  | Recessive | CC + GC | GG | 0.374 (0.014–9.656) | 0.424 | – | – | – | – |
|  | Overdominant | CC + GG | GC | 1.442 (0.672–3.094) | 0.206 | 1.340 (0.381–4.716) | 0.539 | 1.025 (0.251–4.187) | 0.963 |
| rs14063 G > A | Co-dominant | GG | AG | 1.410 (0.798–2.490) | 0.111 | 1.698 (0.591–4.882) | 0.186 | 2.014 (0.802–5.058) | 0.045 |
|  |  |  | AA | 1.739 (0.550–5.503) | 0.205 | 0.768 (0.107–5.507) | 0.723 | 1.507 (0.343–6.629) | 0.465 |
|  | Dominant | GG | AG + AA | 1.451 (0.839–2.508) | 0.073 | 1.511 (0.545–4.185) | 0.285 | 1.900 (0.798–4.525) | 0.051 |
|  | Recessive | GG + AG | AA | 1.512 (0.490–4.671) | 0.333 | 0.578 (0.088–3.793) | 0.442 | 1.094 (0.264–4.533) | 0.868 |
|  | Overdominant | GG + AA | AG | 1.324 (0.761–2.304) | 0.182 | 1.774 (0.647–4.866) | 0.134 | 1.875 (0.776–4.532) | 0.060 |
| rs2273068 C > T | Co-dominant | CC | CT | 1.058 (0.526–2.128) | 0.831 | 0.524 (0.131–2.100) | 0.220 | 1.235 (0.418–3.651) | 0.607 |
|  |  |  | TT | 0.919 (0.021–39.714) | 0.953 | – | – | 1.403 (0.031–63.122) | 0.815 |
|  | Dominant | CC | CT + TT | 1.052 (0.529–2.095) | 0.845 | 0.491 (0.124–1.949) | 0.174 | 1.245 (0.434–3.577) | 0.583 |
|  | Recessive | CC + CT | TT | 0.911 (0.021–39.217) | 0.948 | – | – | 1.352 (0.030–60.538) | 0.834 |
|  | Overdominant | CC + TT | CT | 1.057 (0.527–2.118) | 0.834 | 0.535 (0.134–2.139) | 0.234 | 1.230 (0.417–3.628) | 0.614 |
| rs73733015 C > G | Co-dominant | CC | CG | 0.923 (0.508–1.678) | 0.725 | 0.641 (0.219–1.881) | 0.276 | 0.495 (0.194–1.264) | 0.048 |
|  |  |  | GG | 0.751 (0.175–3.216) | 0.603 | – | – | 0.449 (0.051–3.964) | 0.332 |
|  | Dominant | CC | CG + GG | 0.900 (0.507–1.599) | 0.630 | 0.551 (0.191–1.588) | 0.137 | 0.490 (0.198–1.210) | 0.037 |
|  | Recessive | CC + CG | GG | 0.764 (0.181–3.222) | 0.621 | – | – | 0.572 (0.067–4.855) | 0.491 |
|  | Overdominant | CC + GG | CG | 0.938 (0.519–1.695) | 0.776 | 0.714 (0.246–2.073) | 0.404 | 0.524 (0.208–1.316) | 0.064 |

Analysis was conducted after adjustment for covariates, including age, gender and ethnicity. OR, odds ratio; confidence interval, Bonferroni correction confidence interval; ^*^P-value < 0.00833 indicated a statistically significant difference after Bonferroni correction (shown in bold; 6 SNPs = 6 tests).
