# Supplementary material for: Associations of TFEB Gene Polymorphisms With Cognitive Function in Rural Chinese Population
Source: Front Aging Neurosci. 2021 Dec 14;13:757992. doi: 10.3389/fnagi.2021.757992 (PMC8713571; doi:10.3389/fnagi.2021.757992)
Supplement: Supplementary file 3 [file Table_3.docx]

TABLE S3 | Associations between genetic models of the four SNPs and [cognition](D:/360%E4%B8%8B%E8%BD%BD/Youdao/Dict/8.9.8.0/resultui/html/index.html#/javascript:;) [impairment](D:/360%E4%B8%8B%E8%BD%BD/Youdao/Dict/8.9.8.0/resultui/html/index.html#/javascript:;), stratified by ethnicity.

| SNP | Model | Genotype | | Yao minority | | Others | |
| --- | --- | --- | --- | --- | --- | --- | --- |
|  |  | Reference | Alternate | Adjusted OR  (confidence interval) | ^*^P | Adjusted OR  (confidence interval) | ^*^P |
| rs1015149 C > T | Co-dominant | CC | CT | 0.986 (0.562–1.730) | 0.948 | 1.865 (0.766–4.540) | 0.065 |
|  |  |  | TT | 1.352 (0.658–2.778) | 0.269 | 3.105 (0.913–10.566) | 0.015 |
|  | Dominant | CC | CT + TT | 1.080 (0.638–1.830) | 0.699 | 2.123 (0.914–4.932) | 0.018 |
|  | Recessive | CC + CT | TT | 1.363 (0.718–2.587) | 0.202 | 2.185 (0.716–6.669) | 0.065 |
|  | Overdominant | CC + TT | CT | 0.885 (0.537–1.460) | 0.520 | 1.299 (0.585–2.883) | 0.387 |
| rs1062966 C > T | Co-dominant | CC | CT | 0.563 (0.317–0.999) | 0.008 | 0.841 (0.350–2.020) | 0.602 |
|  |  |  | TT | 0.347 (0.065–1.847) | 0.095 | 0.640 (0.041–997) | 0.669 |
|  | Dominant | CC | CT + TT | 0.543 (0.311–0.946) | 0.004 | 0.826 (0.351–1.943) | 0.555 |
|  | Recessive | CC + CT | TT | 0.412 (0.078–2.167) | 0.159 | 0.677 (0.044–10.366) | 0.706 |
|  | Overdominant | CC + TT | CT | 0.586 (0.332–1.035) | 0.013 | 0.853 (0.357–2.039) | 0.631 |
| rs11754668 C > G | Co-dominant | CC | GC | 1.250 (0.644–2.426) | 0.374 | 1.131 (0.378–4.980) | 0.518 |
|  |  |  | GG | 2.004 (0.073–54.666) | 0.579 | – | – |
|  | Dominant | CC | GC + GG | 1.271 (0.661–2.442) | 0.333 | 1.234 (0.352–4.327) | 0.659 |
|  | Recessive | CC + GC | GG | 1.932 (0.071–52.645) | 0.599 | – | – |
|  | Overdominant | CC + GG | GC | 1.244 (0.642–2.413) | 0.384 | 1.390 (0.383–5.045) | 0.501 |
| rs14063 G > A | Co-dominant | GG | AG | 1.439 (0.855–2.423) | 0.065 | 2.204 (0.942–5.158) | 0.014 |
|  |  |  | AA | 1.281 (0.487–3.368) | 0.500 | 1.899 (0.439–8.225) | 0.248 |
|  | Dominant | GG | AG + AA | 1.415 (0.858–2.332) | 0.067 | 2.147 (0.958–4.812) | 0.012 |
|  | Recessive | GG + AG | AA | 1.077 (0.423–2.741) | 0.833 | 1.376 (0.328–5.771) | 0.557 |
|  | Overdominant | GG + AA | AG | 1.399 (0.844–2.320) | 0.080 | 1.997 (0.881–4.529) | 0.026 |
| rs2273068 C > T | Co-dominant | CC | CT | 0.991 (0.516–1.903) | 0.970 | 0.991 (0.383–2.567) | 0.980 |
|  |  |  | TT | 0.794 (0.030–20.673) | 0.852 | 0.943 (0.021–41.989) | 0.967 |
|  | Dominant | CC | CT + TT | 0.983 (0.517–1.871) | 0.945 | 0.989 (0.389–2.513) | 0.974 |
|  | Recessive | CC + CT | TT | 0.793 (0.031–20.595) | 0.851 | 0.944 (0.021–41.874) | 0.968 |
|  | Overdominant | CC + TT | CT | 0.992 (0.517–1.906) | 0.975 | 0.992 (0.383–2.566) | 0.981 |
| rs73733015 C > G | Co-dominant | CC | CG | 0.733 (0.427–1.259) | 0.130 | 0.835 (0.359–1.942) | 0.572 |
|  |  |  | GG | 0.686 (0.171–2.748) | 0.474 | 0.325 (0.045–2.366) | 0.135 |
|  | Dominant | CC | CG + GG | 0.728 (0.432–1.228) | 0.110 | 0.749 (0.333–1.688) | 0.348 |
|  | Recessive | CC + CG | GG | 0.756 (0.190–3.004) | 0.593 | 0.325 (0.047–2.266) | 0.127 |
|  | Overdominant | CC + GG | CG | 0.747 (0.437–1.277) | 0.151 | 0.911 (0.398–2.088) | 0.768 |

Analysis was conducted after adjustment for covariates, including age, gender and education level. OR, odds ratio; confidence interval, Bonferroni correction confidence interval; ^*^P-value < 0.00833 indicated a statistically significant difference after Bonferroni correction (shown in bold; 6 SNPs = 6 tests).
